# EZH2 suppression in glioblastoma shifts microglia toward M1 phenotype in tumor microenvironment

**DOI:** 10.1186/s12974-017-0993-4

**Published:** 2017-11-13

**Authors:** Yatao Yin, Shuwei Qiu, Xiangpen Li, Bo Huang, Yun Xu, Ying Peng

**Affiliations:** 1 Department of Neurology, Sun Yat-sen Memorial Hospital, Sun Yat-sen University, Yangjiang Xi Road 107, Guangzhou, China; 2 Department of Neurology, Affiliated Drum Tower Hospital of Nanjing University Medical School, Zhongshan Road 321, Nanjing, China; 3 Department of Rehabilitation Medicine, Tongji Hospital, Tongji Medical College, Huazhong University of Science and Technology, Wuhan, China; 4 Guangdong Provincial Key Laboratory of Malignant Tumor Epigenetics and Gene Regulation, Sun Yat-Sen Memorial Hospital, Sun Yat-sen University, Guangzhou, China

**Keywords:** Enhancer of zeste homolog 2 (EZH2), Glioblastoma, Microglia, Polarization

## Abstract

**Background:**

Glioblastoma multiforme (GBM) induces tumor immunosuppression through interacting with tumor-infiltrating microglia or macrophages (TAMs) with an unclear pathogenesis. Enhancer of zeste homolog 2 (EZH2) is abundant in GBM samples and cell lines and is involved in GBM proliferation, cell cycle, and invasion, whereas its association with innate immune response is not yet reported. Herein, the aim of this study was to investigate the role of EZH2 in GBM immune.

**Methods:**

Co-culturing models of human/murine GBM cells with PBMC-derived macrophages/primary microglia were employed. EZH2 mRNAs and function were suppressed by siEZH2 and DZNep. Real-time PCR and flow cytometry were used to determine levels of microglia/macrophages markers. The fluorescence-labeled latex beads and flow cytometry were utilized to evaluate phagocytic abilities of microglia. CCK8 assay was performed to assess microglia proliferation.

**Results:**

EZH2 inhibition led to significant reduction of TGFβ1-3 and IL10 and elevation of IL1β and IL6 in human and murine GBM cells. More importantly, EZH2 suppression in GBM cells resulted in significant increase of M1 markers (TNFα and iNOS) and decrease of a pool of M2 markers in murine microglia. The proportion of CD206^+^ cells was decreased in PBMC-derived macrophages as co-incubated with EZH2-inhibited GBM cells. Functional researches showed that phagocytic capacities of microglia were significantly ameliorated after EZH2 inhibition in co-culturing GBM cells and microglia proliferation was declined after addition of TGFβ2 antibodies to co-incubated GBM cells with EZH2 inhibition. Besides, we found that EZH2 suppression in GBM cells enhanced co-culturing microglia engulfment through activation of iNOS.

**Conclusions:**

Our data demonstrates that EZH2 participates in GBM-induced immune deficient and EZH2 suppression in GBM can remodel microglia immune functions, which is beneficial for understanding GBM pathogenesis and suggests potential targets for therapeutic approaches.

**Electronic supplementary material:**

The online version of this article (10.1186/s12974-017-0993-4) contains supplementary material, which is available to authorized users.

## Background

Glioblastoma multiforme (GBM) is the most aggressive malignancy among primary brain tumors with very low median survival. Due to the feature of high heterogeneity and infiltration, GBM’s etiology and pathophysiologic mechanisms remain unclear, resulting in limited treatment approaches. However, mounting evidence demonstrates that immune response is involved in development and progression of GBM [1], and therefore, thorough investigation of the underlying molecular and immunologic mechanism will be beneficial for novel interventions.

GBMs are complex solid tumors containing neoplastic and non-neoplastic cells. The majority of the non-neoplastic cells are tumor-associated macrophages (TAMs), either of peripheral origin or brain resident microglia, which account for 30% of total tumor mass and are reported to play several roles in GBM progression including proliferation, motility, survival, and immunosuppression [1, 2]. TAMs are recruited to GBM environment and release a wide array of chemokines and cytokines in response to those factors produced by GBMs [1]. As a result, TAMs facilitate tumor proliferation, survival, and migration, acting as an anti-immunological and pro-tumorigenic role in the tumor microenvironment.

Microglia/macrophages exist in a spectrum of phenotypes. The classically activated microglia/macrophages, M1 phenotype, stimulate anti-tumor immune response through secretion of pro-inflammatory cytokines, such as IFN-gamma, IL1β, iNOS, etc., whereas the alternatively activated or M2 phenotypes promote tumor survival via producing anti-inflammatory cytokines like IL4, TGFβ, IL10, etc. [3]. Within the tumor microenvironment, TAMs are forced forwards M2 phenotypes by GBM cells via secreting a wide variety of factors such as IL10, IL4, IL6, M-CSF, macrophage inhibitory factor (MIF), TGFβ, and prostaglandin E2 (PGE2), and subsequently support tumor growth and invasion [4]. However, the pathogenesis of elevation of these anti-immune factors and reduction of pro-immune factors in GBM remains elucidated.

Enhancer of zeste homolog 2 (EZH2) is the core catalytic subunit of Polycomb repressive complexes 2 (PRC2) and can silence a bundle of tumor suppressor genes through methylation of lysine 27 of histone 3 (H3K27) of target genes [5]. It has been reported to aberrantly express in multiple solid tumors. In particular, accumulated data including our study verify that EZH2 is highly abundant in GBM samples and that expression levels of EZH2 are positively correlated with GBM grades and unfavorable survival [6, 7], which is therefore suggested as a biomarker for diagnosis and prognosis. In our previous study, we demonstrate EZH2 promotes GBM cell proliferation, cell cycle progression, inhibition of apoptosis, and invasion, serving as an oncogene [8]. Recently, EZH2 is reported to be involved in adaptive immune response, whereas little information about EZH2 in innate immune response is available, especially in GBM.

In the present study, we aimed to investigate the influence of EZH2 on immune functions of microglia or macrophages in a co-culturing model with GBM cells. We demonstrated that EZH2 inhibition in GBM induced elevation of M1 makers (iNOS and TNFα) and reduction of a pool of M2 markers in murine microglia and human PBMC-derived macrophages. Furthermore, we found that EZH2 inhibition in GBM cells enhanced phagocytic capacities of co-culturing microglia through activation of iNOS.

## Methods

### Isolation of human monocyte-derived macrophages

Human peripheral blood mononuclear cells (PBMCs) were isolated using sequential Ficoll and Percoll density gradient centrifugations as described previously [9]. Blood samples were collected from healthy volunteers, and the procedure was approved by the ethics committee of Sun Yat-sen Memorial Hospital, Sun Yat-sen University. Monocytes were purified using CD14 microbeads according to the manufacturer’s directions (Miltenyi Biotec) and then were treated with 20 ng/ml macrophage colony stimulating factor (M-CSF, switching monocytes to M2 phenotype macrophages) and cultured at 37 °C and 5% CO_2_ in ultra-low attachment flasks (Corning) for 5 day in the RPMI 1640 medium with 10% fetal bovine serum (FBS, Gibco).

### Isolation and culture of microglia

Primary microglia were prepared and isolated using 1-day-old C57BL/6 mice as described previously [10]. Briefly, cells were isolated by trypsinization, mechanically dissociated and plated into 75-cm^2^ flasks (BD Falcon) pre-coated with 0.4 μg/ml poly-L-lysine (Sigma-Aldrich) in DMEM-F12 medium containing 10% FBS, 100 U/mL penicillin and 0.1 mg/mL streptomycin (Invitrogen). Cells were maintained at 37 °C in a humidified atmosphere with 5% CO_2_. The culture medium was changed after 1 day and then twice a week. Ten to fourteen days later, floating and loosely attached microglia were manually shaken off, were centrifuged (200 g, 6 min), and were suspended in the culture medium and plated at a final density of 2–3 × 10^5^ cells/ml. Adherent cells (96% positive for microglia marker Iba1) were incubated for 48 h prior to experiments.

### Cell culture and co-culture of GBM with macrophage/microglia

Human U87 and U251 GBM cell lines were obtained from China Academia Sinica cell repository (Shanghai, China). Cells were maintained in Dulbecco’s modified Eagle’s medium (DMEM, Hyclone) supplemented with 10% FBS and incubated at 37 °C with 5% CO_2_. Murine GL261 GBM cell line was obtained from the Clinical Research Center of Nan-Fang Hospital of Southern Medical University (Guangzhou, China) and were cultured in DMEM-F12 medium containing 10% FBS.

Co-cultivation of PBMC-derived macrophages or mouse primary microglia with GBM cells (U87, U251, or GL261) were performed in 12-well Boyden chambers (Corning). GBM cells were seeded on the 0.4-μM inserts, which are permeable to supernatants but not to cellular components, with the cell density of 1 × 10^5^ cells/insert. Macrophages or microglia were seeded in the lower chambers (5 × 10^4^ cells/cm^2^) and grown for indicated periods of time.

GBM conditioned medium (GCM) was collected after 24 h culture and applied to microglia in a volume of 2 ml (35-mm dish) or 100 μl (96-well plate). The fresh medium was used as controls.

### EZH2 siRNA transfection and EZH2 function inhibition

EZH2 siRNA (siEZH2) and negative control (NC) oligonucleotides were purchased from GenePharma (Shanghai, China, see Additional file 1). Oligonucleotides were transected into U87, U251, and GL261 cells using the transfection reagent Lipofectamine2000 (Invitrogen) following the manufacturer’s instructions with oligonucleotides at a working concentration of 100 nM. The transfected cells were incubated for 24 or 48 h before subsequent experiments.

The EZH2 functional inhibitor, DZNep, was purchased from Sigma-Aldrich. DZNep was added to U87 and U251 cell medium at a concentration of 5 μM for indicated time duration.

### Gene expression microarray and bioinformatics analysis

GBM sample preparation: U87 cells were treated with siEZH2 and NC for 48 h and then total RNA was extracted using a TRIzol reagent (Life Technologies, USA) according to the manufacturer’s instructions. The same samples were repeated twice and then these three copy samples were mixed for next processes.

Microarray: The quantification and quality were assessed by spectrophotometry and an Agilent 2100 Bioanalyzer (Agilent Technologies, USA). The RNA samples at 2 μg without degradation was amplified, labeled, and hybridized. Gene expression profile was analyzed using the Agilent Whole Human Genome Oligo Microarray kit, 4 × 44 K (Agilent Technologies, USA), consisting of approximately 41,000 genes and transcripts. Ratio was calculated by comparison between siEZH2 and NC group. The experimental data were analyzed using the SBC Analysis System, including gene ontology (GO) and pathway enrichment analysis. Biological processes terms were performed by a functional enrichment analysis tool, FunRich_V3 (http://www.funrich.org/).

### RNA extraction and quantitative real-time PCR

Total RNA was extracted using Trizol reagent (Invitrogen) according to its protocol and was reversely transcribed using PrimeScript RT Reagent Kit (Takara, Otsu, Shiga, Japan). Quantitative real-time PCR (qPCR) was performed using SYBR Green PCR master mix (Takara) on an Applied Biosystems StepOnePlus™ System (Applied Biosystems, Foster City, CA, USA). All qPCR reactions were performed in triplicate, and relative quantifications (RQs) were calculated using the 2^-△△Ct^ method. The sequences of PCR primers were listed in Additional file 1.

### Protein isolation and western blotting

Cells were washed twice with ice-cold PBS, and lysed in RIPA buffer (Pierce, Waltham, MA, USA). Protein lysates were separated by 8–10% SDS-PAGE and then were electrophoretically transferred to PVDF membrane (Millipore, Lake Placid, NY, USA). After blocked in 5% non-fat milk, the membrane was incubated with either rabbit anti-human EZH2 (1:1000, Cell Signaling Tech., Beverly, MA, USA), rabbit anti-human GAPDH antibody (1:1000, CST), followed by HRP (horseradish peroxidase)-labeled goat-anti-mouse or goat-anti-rabbit IgG (1:5000, Santa Cruz). Protein levels were detected using ECL detection solution (Pierce) and visualized on Bio-Rad ChemiDocXRS (Bio-Rad Laboratories, Hercules, CA, USA).

### Phagocytosis assay

Microglia were plated onto 35-mm dishes at a density of 2 × 10^5^/ml and were co-incubated with different conditions of GBM cells for 24 h. Then the fluorescence-labeled latex beads (Sigma) were added at the concentration of 4 μl/ml for 90 min at 37 °C. Later, cells were washed three times with PBS to remove non-phagocytized beads and were fixed with 4% paraformaldehyde. Next, bead phagocytosis of microglia was observed under confocal microscope (Olympus fv10i). In order to quantify phagocytosis, cell numbers with low (< 2 beads/cell), medium (2–10 beads/cell), or high (≥ 10 beads/cell) phagocytic activity were counted using a fluorescent microscope (× 20 magnification). In addition, murine GL261 cells were pre-treated with siEZH2 and DZNep for 24 h, and then, GL261 were removed and 1400 W (a specific iNOS inhibitor, Sigma) was added to microglia for another 24 h with a working concentration of 500 μM. Next, fluorescent latex beads were added to microglia for 90 min. Amounts of latex beads phagocytized by microglia were detected by flow cytometry. All these experiments were repeated thrice.

### Flow cytometry

PBMC-derived macrophages co-cultured with U87 cells for 24 h were washed and resuspended in PBS. For intracellular markers, cells were fixed and permeabilized with permeabilization reagents (CALTAGTM Laboratories) and then incubated with anti-human CD68-FITC (macrophage marker, BD Bioscience, CAS: 562117) or anti-human CD206-APC (M2 microglia marker, BD Bioscience, CAS: 561763). After the final washing step, cells were analyzed by flow cytometry (BD). Flow cytometry was also used to detect microglia amounts containing fluorescent beads, as mentioned above.

### Nitric oxide production

Murine primary microglia were co-cultured with GBM cells GL261 with different treatments for 24 h. Aliquots of cell culture supernatant were taken at 24 h for total NO production by using the Griess assay according to the protocol of the manufacturer (Beyotime Institute Biotechnology, Jiangmen, China), as absorbance at 540 nm from the ELISA plate reader [11]. The amount of nitrite was normalized to the amount produced by untreated microglia.

### Cell proliferation assay

Primary microglia were seeded in a 96-well culture plates at 2 × 10^4^ cells/well in 100 μl for 48 h to achieve resting state. The medium was then replaced with 100 μl conditioned medium from GL261 cells, which were pre-treated with siEZH2 and DZNep with working concentrations of 100 nM and 5 μM, respectively. 24 h later, cell proliferation was determined by the CCK-8 assay Kit. Additionally, cytokines TGFβ1(Peprotech, CAS100-21), TGFβ2 (Peprotech, 100-35B), and 1400 W (an iNOS inhibitor, sigma) were added to primary microglia for 24 h at working concentrations of 10 μg/ml, 10 μg/ml, and 500 uM followed by CCK-8 assay. Besides, PBS were used as control. The absorbance of the solution was measured on a SpectraMax M5 multimode microplate reader at 450 nm. Cell viability of each group was expressed as a percentage relative to control.

### Statistical analysis

All quantified data were repeated thrice at least and expressed as mean ± SD. Comparison between groups was analyzed by one-way analysis of variance (ANOVA) followed by Bonferroni post hoc test. *P* < 0.05 was set as statistical significance.

## Results

### EZH2 inhibition in GBM cells is associated with immune responses

Initially, global gene expression pattern related with EZH2 inhibition by siRNAs in GBM U87 cell line was investigated using gene microarray assay. We found that EZH2-related genes were enriched in KEGG *TNF signaling pathway* and biological process *immune response* (see Additional file 2), which suggested that EZH2-associated genes played roles in immune response in GBM cells.

**Fig. 1:**
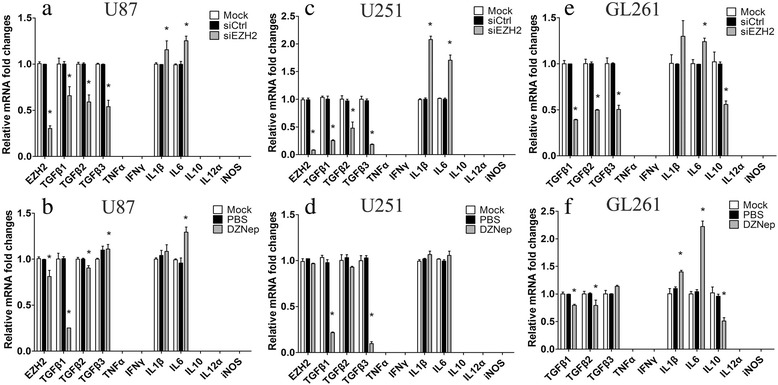
EZH2 inhibition induces mRNAs changes of inflammatory cytokines in GBM cells. **a** and **b** Human U87 cells were treated with siEZH2 and DZNep for 24 h, and then, mRNA levels of several cytokines were determined by quantitative real-time PCR. **c** and **d** Human U251 cells were treated similar to U87, and mRNA expression of several cytokines were determined by qPCR. **e** and **f** Murine GL261 cells were treated similar to U87, and mRNA expression of several cytokines were determined by real time PCR. NC and PBS were set as controls, respectively, and nothing were added to mock group. All these experiments were repeated thrice. *p < 0.05, compared with corresponding control group

Then, expression levels of these immune and inflammation genes were verified in human U87 and U251 and murine GL261 GBM cell lines. Three independent murine siEZH2 were compared by qPCR and western blot (see Additional file 3). Then we chose the one due to its potent inhibition on EZH2 expression for the subsequent studies in GL261 glioma cell lines. The qPCR results showed that knockdown of EZH2 in GBM cells induced decline of TGFβ1-3 and IL10 and elevation of IL1β and IL6 (Fig. 1). Consistent with alternation patterns of siEZH2-induced cytokines, addition of DZNep (an EZH2 functional inhibitor) also resulted in the similar change of these immune cytokines (Fig. 1). It should be noted that mRNA levels of some cytokines such as IL12α, TNFα, IFNγ, and iNOS are too low to detect in three GBM cell lines by qPCR (Fig. 1). These data suggested that EZH2 was indeed involved in GBM immune response.

### EZH2 suppression in GBM cells switches microglia polarization toward M1 phenotype

**Fig. 2:**
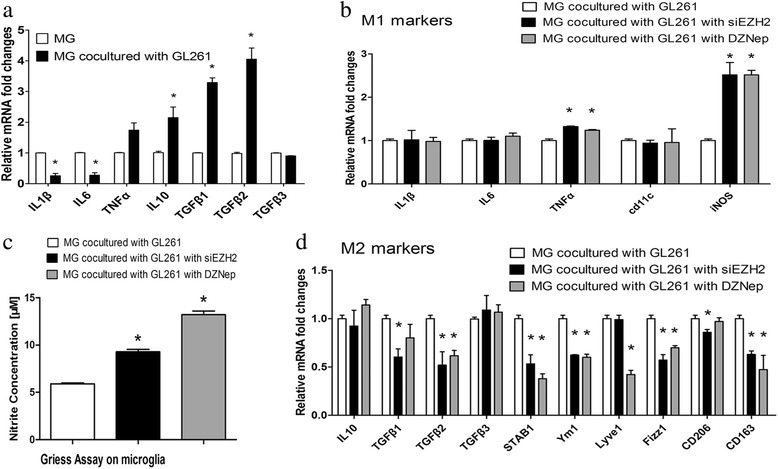
EZH2 suppression in GBM cells switches microglia polarization toward M1 phenotypes. **a** Murine primary microglia (PM) were co-cultured with GL261 cells for 24 h, and mRNA expression of multiple cytokines of microglia were determined by real-time PCR. **b** GL261 cells were treated with siEZH2 and DZNep for 24 h and then co-cultured with PM cells for another 24 h. Next, mRNAs of several M1 markers of microglia were tested by qPCR. **c** GL 261 cells were treated similar to **b**. Then, NO production in supernatant of the co-culture system was defined using the Griess assay. The amount of nitrite was normalized to untreated PM. **d** GL261 cells were treated similar to **b**, and mRNAs of multiple M2 markers were tested by qPCR. NC and PBS were set as controls, respectively, and nothing were added to mock group. All these experiments were repeated thrice. *p < 0.05, compared with corresponding control group

**Fig. 3:**
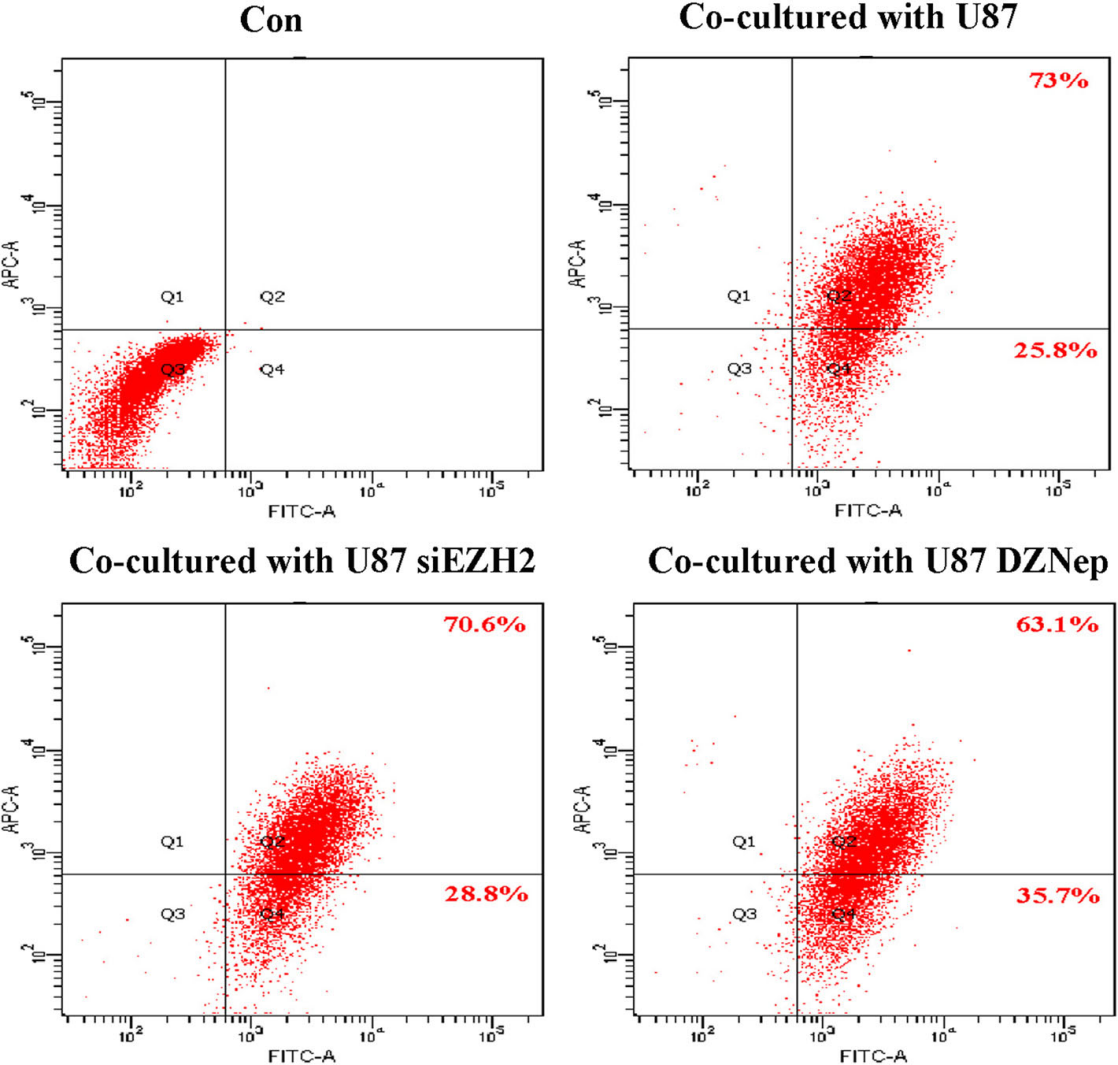
EZH2 inhibition in GBM cells weakens M2 phenotype of PBMC-derived macrophages. Human U87 GBM cells were incubated with siEZH2 and DZnep for 24 h and then co-cultured with PBMC-derived macrophages of M2 phenotype prior induced by cytokines M-CSF. Another 24 h later, macrophages were harvested for evaluation of CD206 (an M2 marker) by flow cytometry. Right half quadrant shows macrophages, whereas right upper shows M2 phenotype of macrophages. NC and PBS were set as controls and just medium were added to mock group. This experiment was repeated twice, and one representative result was illustrated

Next, we attempted to explore influences of EZH2 on microglia polarization. First, using GBM-microglia/macrophages co-culturing models, we show that pro-inflammatory cytokines (IL1β and IL6) were decreased and anti-inflammatory cytokines (TGFβ1, TGFβ2, and IL10) were increased in microglia treated with murine GL261 cells (Fig. 2a). Similarly, amounts of PBMC-derived macrophages labeled by CD206 were significantly enhanced after co-incubation with human U87 cells (Fig. 3b). These data indicated that GBM cells were able to switch microglia or macrophage toward the anti-inflammatory M2 type.

Then, we intended to find out that whether inhibition of EZH2 expression and function would reverse the above phenotype changes of microglia/macrophage induced by GBM. The results showed that significant increase of TNFα and iNOS were observed with no change of IL1β, IL6, and CD11c in microglia co-cultivated with murine GBM cells pre-treated with siEZH2 and DZNep (Fig. 2b). Accordant with the mRNA expression, endogenous NO production from microglia was markedly increased as well (Fig. 2c). By contrast, a pool of M2 markers (TGFβ1-2, STAB1, Ym1, Lyvel, Fizz1, CD206, CD163) were declined (Fig. 2d) in murine microglia. In human co-culture models, proportions of CD206^+^ cells were decreased to 70.6 and 63.3% in PBMC-derived macrophages co-cultured with human EZH2-inhibited U87 cells by siEZH2 and DZNep, respectively (Fig. 3c, d). Taken together, these data suggested that EZH2 inhibition of GBM cells could switch co-culturing microglia or macrophages polarization from M2 toward M1 phenotypes.

### EZH2 inhibition in GBM cells ameliorates microglia phagocytosis with iNOS dependent

**Fig. 4:**
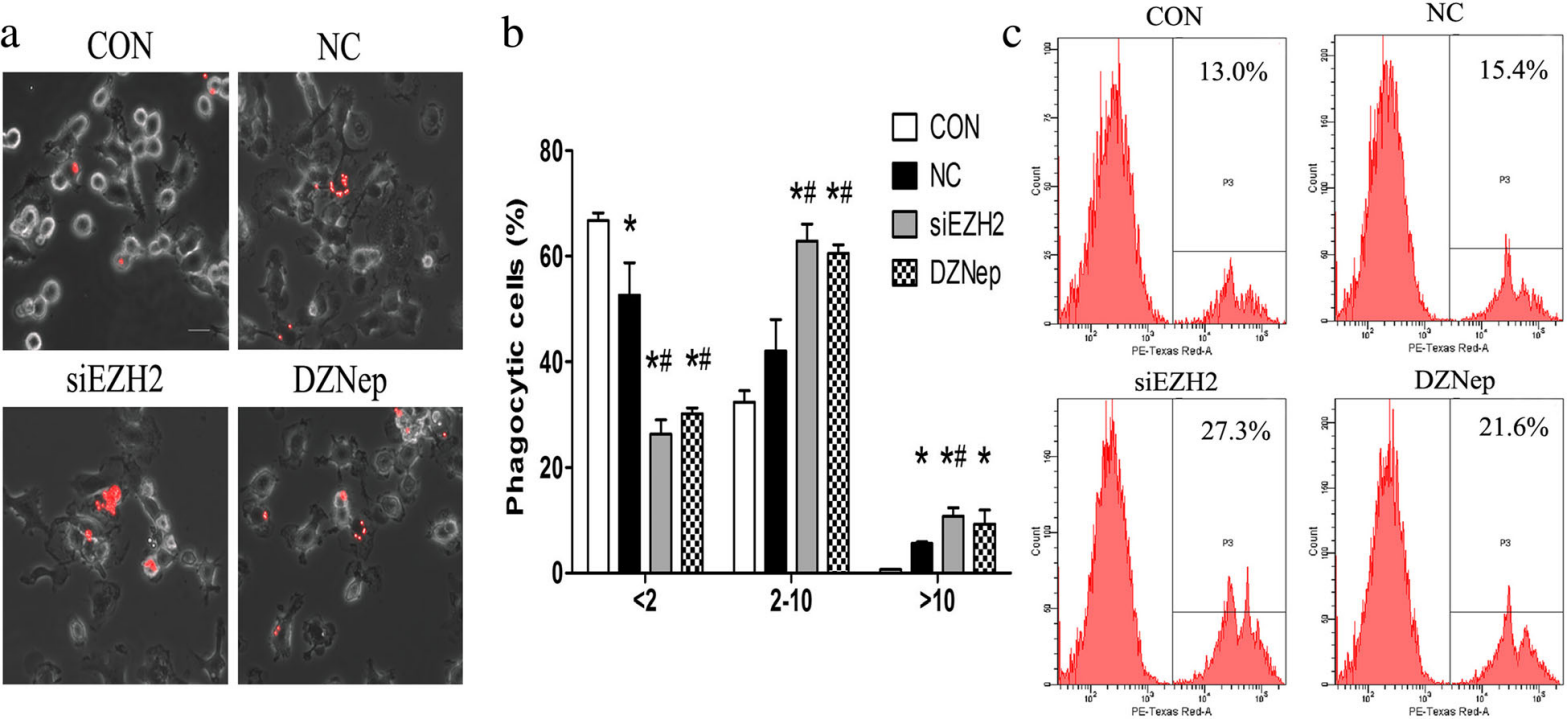
Downregulation of EZH2 in GBM enhances microglia phagocytosis. **a** Murine GL261 cells were pre-treaded with siEZH2 and DZNep for 24 h and then co-cultured with primary microglia (PM) for another 24 h. The fluorescent latex beads were added to microglia for 90 min. Then, the phagocytosis of microglia, indicated as red fluorescence, was observed by confocal microscope. Representative pictures of phagocytic microglia were illustrated; scale bar = 20 μm. **b** Quantification of PM amounts with phagocytic activity as shown in **a**. Microglia were divided into three groups in virtue of fluorescent beads contained within PM (< 2 beads/cell, 2–10 beads/cell, and > 10 beads/cell) in 20 randomly selected fields. Data are presented as the mean ± SD from three experiments performed on independently derived microglia cultures. **c** Murine GL261 cells were pre-treaded with siEZH2 and DZNep for 24 h and then co-cultured with primary microglia for another 24 h. The fluorescent latex beads were added to microglia for 90 min. Amounts of latex beads phagocytized by microglia were detected by flow cytometry. NC and PBS were set as controls, respectively, and nothing were added to Con group. All these experiments were repeated thrice. *p < 0.05 vs. MG group; #*p* < 0.05 vs. GCM group

To evaluate the role of EZH2 in microglia phagocytosis, microglia were co-cultured with EZH2-inhibited GL261 cells by siEZH2 or DZNep, and microglia engulfment of fluorescent beads were determined by the confocal microscopy. Compared with controls, the amounts of beads-containing cells from GL261-conditioned microglia were mildly increased (Fig. 4a). However, as co-cultured with EZH2-inhibited GL261, microglia phagocytized much more latex beads (Fig. 4a). Likewise, microglia containing latex beads were counted under fluorescent microscope, and the data showed that amounts of microglia engulfing more than two beads were higher than controls, even though microglia containing less than two beads were reduced in microglia co-cultured with EZH2-inhibited GL261 (Fig. 4b). This suggested that microglia phagocytosis capacity was improved as EZH2 was inhibited in co-incubated GBM cells.

At the same time, microglia phagocytosis capacity was assessed by flow cytometry. Compared to resting microglia (13.0% ± 0.1%), engulfment of fluorescent beads were significantly amplified in microglia co-cultured with EZH2-inhibited GL261 (27.5% ± 0.3% for siEZH2; 21.2% ± 0.5% for DZNep; *p* < 0.05), while GL261 conditioned microglia did not change phagocytic ability (Fig. 4c).

**Fig. 5:**
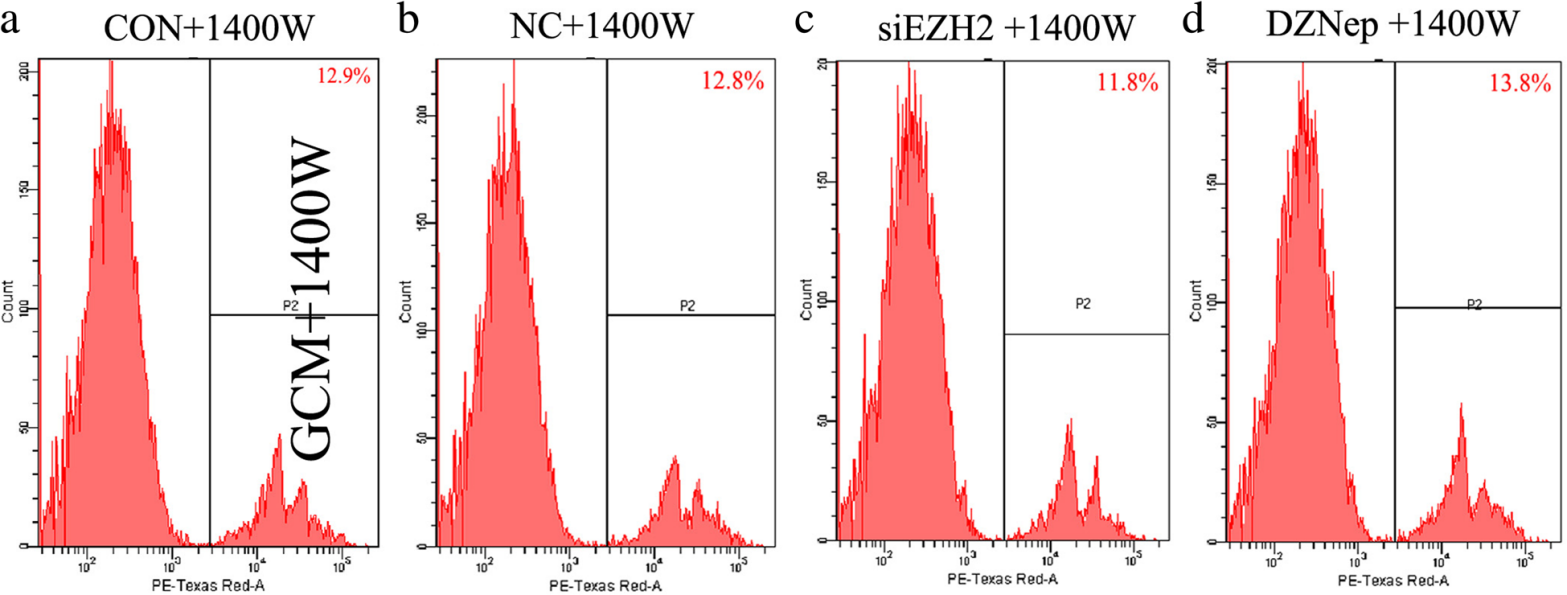
EZH2 inhibition in GBM stimulates phagocytosis in microglia in an iNOS-dependent manner. **a**-**d** Murine GL261 cells were pre-treated with siEZH2 and DZNep for 24 h, and then, GL261 were removed and 1400 W, a specific iNOS inhibitor, was added to microglia for another 24 h with a working concentration of 500 μM. Next, fluorescent latex beads were added to microglia for 90 min. Amounts of latex beads phagocytized by microglia were detected by flow cytometry. NC was used as controls, and nothing was added to CON group. The experiments were repeated thrice, and one representative experiment was shown here

Given the high rise of iNOS expression and NO production in microglia co-cultured with EZH2-suppressed GBM cells, we wondered whether iNOS was involved in microglia phagocytosis. Initially, microglia were co-cultured with EZH2-suppressed GL261 cells and then GL261 were removed and 1400 W, a specific iNOS inhibitor, was added to microglia for 24 h. Then, by virtue of fluorescent beads, the phagocytosis of microglia were observed. The results showed that in comparison to controls, untreated GL261 did not change the phagocytic ability, while the phagocytic ability of microglia did not change in microglia treated with EZH2-suppressed GBM, either (Fig. 5). These data strongly suggested that EZH2 inhibition in GBM ameliorated microglia phagocytosis through activation of iNOS.

### EZH2 inhibition in GBM promotes microglia proliferation

**Fig. 6:**
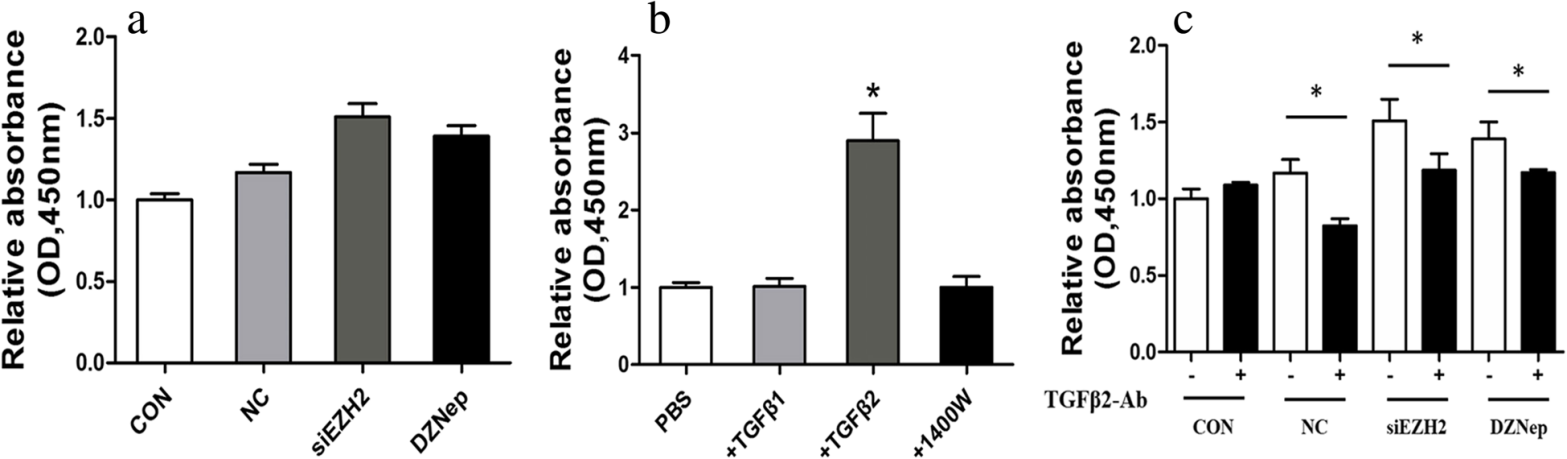
GBM cells with EZH2 inhibition can promote microglia proliferation with TGFβ2 dependent. **a** Murine GL261 cells were treated with siEZH2 and DZNep for 24 h and then co-incubated with primary microglia for another 24 h. Next, microglia proliferation was determined by CCK-8 assays. **b** Primary microglia were incubated with cytokines TGFβ1, TGFβ2, and 1400 W with working concentrations of 10 μg/ml, 10 μg/ml, and 500 uM. Twenty-four hours later, cell proliferation was analyzed by CCK-8 assays. **c** Murine GL261 cells were treated with siEZH2 and DZNep for 24 h and then co-incubated with primary microglia for another 24 h. Next, GBM were removed and TGFβ2 antibodies were added into microglia at the concentration of 10 μg/ml for 24 h. Next, microglia proliferation was analyzed by CCK-8 assays. PBS and NC siRNAs were set as controls for TGFβ2 antibodies and siEZH2, respectively, and nothing were added to CON group. All these experiments were repeated thrice. *p < 0.05

To investigate impacts of EZH2 in GBM cells on microglia proliferation, microglia were co-cultured with GL261 cells pre-treated by siEZH2 or DZNep. We showed that no significant differences were obtained among groups, although an increase trend of microglia proliferation co-culturing with GBM at different conditions (Fig. 6a, ANOVA *p* = 0.058).

Given the significant alternations of several cytokines in GBM and microglia showed in Figs. 1 and 2, their impacts on microglia proliferation were investigated. It was showed that only TGFβ2 but not TGFβ1 and iNOS contributed to obvious microglia proliferation (Fig. 6b). Subsequently, TGFβ2 neutralizing antibodies were added to the co-culturing model of GBM and microglia to verify if they could reverse this effect. We found that microglia proliferation were significantly decreased in antibody addition groups in contrast to the corresponding control groups (Fig. 6c). These data suggested TGFβ2 pathway also played a role in EZH2 mediated GBM action on microglia.

## Discussion

In the present study, we demonstrate that EZH2 plays a role in GBM innate immune response for the first time. We find that EZH2 inhibition in GBM cells enhances phagocytic capacities and viabilities of co-culturing microglia with iNOS and TGFβ2 dependent. Mechanic researches show that knockdown of EZH2 inhibits expression of anti-inflammatory factors while promotes expression of pro-inflammatory factors in GBM cells. More importantly, EZH2 suppression in GBM contributes to polarization shift of microglia and PMMC-derived macrophage, reflected by increase of M1 markers and reduction of M2 markers.

The oncogene EZH2 is of considerable interest as a potential therapeutic target in GBMs. It has been confirmed that EZH2 is highly expressed in GBM samples and cell lines [7, 12], and abundance of EZH2 is associated with high GBM grades and poor survival [6, 7]. Numerous studies report that knockdown of EZH2 by siEZH2 or function suppression by DZNep induce GBM growth inhibition [13–15] and EZH2 is well recognized to take part in multiple tumor processes such as cell cycle [14], proliferation [16], apoptosis [16], invasion and mobility [16], GBM stem cell differentiation and maintenance [17, 18], tumor angiogenesis [19], etc. Our previous study demonstrates that miR-138 effectively inhibits GBM cell proliferation in vitro and tumorigenicity in vivo through directly blocking an EZH2-mediated signal loop [8]. Recently, the relation between EZH2 and immune response is paid more attention. One study reports that EZH2 mediates the humoral immune response and drives lymphomagenesis through formation of bivalent chromatin domains at critical germinal center (GC) B cell promoters [20]. EZH2 is also believed to be involved in T cell function [21]. Another study reports that downregulation of EZH2 results in significant increases in CIITA and HLA-DRA expression as well as increased cell surface expression of MHC II in breast cancer [22]. These studies strongly suggest that EZH2 plays vital roles in immune response and inflammation. However, up to date, no reports were found to focus on EZH2-related immune response in GBM, as we have shown in the present study.

Accumulated evidence demonstrates that there is a strong interaction between GBM and microglia in the tumor microenvironment. It has been confirmed that GBM cells establish an immune-deficit microenvironment to promote TAMs acquiring the M2 phenotype [23]. When microglia polarize to M2 phenotypes, they in turn contribute to local immunosuppression and support GBM growth and migration [23]. To find out exact genes that are changed in TAMs, Szulzewsky et al. used microarray analysis to compare expression profiles of TAMs and naive control cells and demonstrated that expression of Gpnmb and Spp1 are highly upregulated in both murine and human TAMs [24]. However, there are no other studies about these two genes on GBMs.

Our findings indicate that EZH2 inhibition in GBM decreases expression of M2 markers and increases expression of M1 markers in co-culturing microglia. Two of them, iNOS and TGFβ, are altered much more significantly than others. The NOS has three isoforms, including endogenous NOS (eNOS), neuronal NOS (nNOS) and inducible NOS (iNOS), responsible for production of NO from the amino acid L-arginine [25–27]. iNOS are inducible in several types of cells, including epithelial, mesenchymal, and myeloid cells [28], and are reported to be aberrant in various human tumors such as breast and stomach cancer [29–32]. Actually, iNOS has been emphasized in GBM pathogenesis by the fact that inhibition of iNOS by genetic and pharmacological approaches impedes glial cell proliferation, invasiveness, and tumor growth in vivo [33], and that repression of GBM iNOS in vivo leads to a reduction in both microglia recruitment and tumor expansion [34]. iNOS usually exerts a function of promoting microglia/macrophage polarization toward M1 phenotypes. In our study, we show that relative to microglia co-incubated with GBM without intervention, iNOS increases more than 2 times in microglia co-cultured with EZH2-suppressed GBM cells. Together with reductions of other cytokines shown in Fig. 2, these data suggest that iNOS is involved in EZH2-mediated microglia polarization shift.

TGFβ is a multifunctional cytokine that is involved in tissue homeostasis and embryonic development [35, 36]. There are three different isoforms, including TGFβ1, TGFβ2, and TGFβ3. Among them, TGFβ1 and TGFβ2 are involved in brain tumor development and progression, particularly in high-grade GBM [37–41]. Besides, the high activity of the TGF-β signaling pathway in human GBM tissues has been associated with poor prognosis [42]. Our findings demonstrate that EZH2 inhibition in GBM leads to reductions of TGFβ1 and TGFβ2 while TGFβ2 promotes microglia viabilities. This suggests that TGFβ2 is involved in EZH2-mediated GBM progression and growth, which needs more concentration.

Also, we show that EZH2 inhibition in GBM accelerates phagocytosis ability of co-culturing microglia. Ellert-Miklaszewska et al. report primary microglia from rats polarize into alternatively activated cells, as exposed to GBM conditioned medium (GCM) for 6 h [43]. Furthermore, GCM strongly augments motility of microglia in a scratch assay and phagocytosis of fluorescent latex beads [43]. Interestingly, another study shows that microglia are activated after 1 h of co-culture with GBM, but longer in contact with tumor cells appears to silence phagocytic properties of microglia [44]. In this study, we attempt to mimic the real tumor microenvironment and 24-h co-culture is set. Consistent with the above long incubation study, our results show that co-culture of GBM with microglia for 24 h does not change phagocytic ability of microglia. Moreover, we observe that EZH2 inhibition in GBM promotes upregulation of iNOS and production of NO from microglia and improves microglia phagocytosis, which can be reversed by the specific iNOS inhibitor. One recent study has identified a critical role for NO in the microglia phagocytic capacity under inflammatory conditions [45]. They show that both addition of exogenous NO and induction of NO generation improve microglia phagocytic capacity independent of cGMP signaling [45], which is supportive of our results. Therefore, our data provides mechanistic evidence of how EZH2 in GBM influences phagocytic activity of microglia.

## Conclusions

Our data demonstrates that EZH2 plays an important role in GBM-induced immune deficient and suppression of EZH2 in GBM can remodel microglia immune functions, which are beneficial for understanding GBM progression and suggest potential targets for therapeutic approaches.

## Additional files

Additional file 1: The sequences of PCR primers and siRNAs. (DOCX 14 kb)

Additional file 2: The KEGG and biological process enrichment analysis of EZH2-associated genes by microarray. Sample preparation: U87 cells were treated with siEZH2 and NC for 48 h at the concentration of 100 nM and then total RNA was extracted using a TRIzol reagent (Life Technologies, USA) according to the manufacturer’s instructions. The same samples at different times were repeated thrice and then these three copy samples were mixed for microarray using the Agilent Whole Human Genome Oligo Microarray kit, 4 × 44 K (Agilent Technologies, USA), consisting of approximately 41,000 genes and transcripts. The upper shows KEGG pathway and lower shows biological processes. (DOCX 130 kb)

Additional file 3: Expression levels of EZH2 mRNAs and proteins in GL261 glioma cells. The expression levels of EZH2 mRNAs and proteins were detected by real-time PCR and western blot in GL261 glioma cells treated with three kinds of siEZH2 and EZH2 functional inhibitor DZNep for 48 h. The working concentrations of siEZH2 and DZNep were 100 nM and 5 uM, respectively. Due to the potent inhibition on EZH2 expression, siR-419 was chosen for subsequent studies in GL261 glioma cell lines. (DOCX 162 kb)

## Abbreviations

CNS: Central nervous system
eNOS: Endogenous NOS
EZH2: Enhancer of zeste homolog 2
GAMs: GBM-associated microglia/macrophages
GBM: Glioblastoma multiforme
GCM: GBM conditioned medium
GO: Gene ontology
H3K27me: Methylating lysine 27 of histone 3
iNOS: Inducible NOS
M-CSF: Macrophage colony stimulating factor
nNOS: Neuronal NOS
PRC2: Polycomb repressive complexes 2
TCGA: The Cancer Genome Atlas
TMZ: Temozolomide
