# Supplementary material for: EZH2 suppression in glioblastoma shifts microglia toward M1 phenotype in tumor microenvironment
Source: J Neuroinflammation. 2017 Nov 13;14:220. doi: 10.1186/s12974-017-0993-4 (PMC5684749; doi:10.1186/s12974-017-0993-4)
Supplement: Supplementary file 1 — The sequences of PCR primers and siRNAs. (DOCX 14 kb) [file 12974_2017_993_MOESM1_ESM.docx]

**Additional file 1:** The sequences of PCR primers and siRNAs.

| **Gene** | **Sequences** |
| --- | --- |
| Human GAPDH | Forward: 5’- CATGAGAAGTATGACAACAGCCT -3’  Reverse: 5’-AGTCCTTCCACGATACCAAAGT-3’ |
| Human EZH2 | Forward: 5’- ACGGCTTCCCAATAACAGTAG -3’  Reverse: 5’-TGTTTGACACCGAGAATTTGC-3’ |
| Human TGFβ1 | Forward: 5’- CAAGGGCTACCATGCCAACT -3’  Reverse: 5’-AGGGCCAGGACCTTGCTG-3’ |
| Human TGFβ2 | Forward: 5’-CAGCACACTCGATATGGACCA -3’  Reverse: 5’-CCTCGGGCTCAGGATAGTCT-3’ |
| Human TGFβ3 | Forward: 5’-ACTTGCACCACCTTGGACTTC -3’  Reverse: 5’-GGTCATCACCGTTGGCTCA-3’ |
| Human IL1β | Forward: 5’-CCTGTCCTGCGTGTTGAAAGA -3’  Reverse: 5’-GGGAACTGGGCAGACTCAAA -3’ |
| Human IL6 | Forward: 5’-ACTCACCTCTTCAGAACGAATTG-3’  Reverse: 5’-CCATCTTTGGAAGGTTCAGGTTG-3’ |
| MUS GAPDH | Forward: 5’-TGATGACATCAAGAAGGTGGTGAAG-3’  Reverse: 5’-TCCTTGGAGGCCATGTAGGCCAT-3’ |
| MUS EZH2 | Forward: 5’-AGTGACTTGGATTTTCCAGCAC-3’  Reverse: 5’-AATTCTGTTGTAAGGGCGACC-3’ |
| MUS TGFβ1 | Forward: 5’-ACTGGAGTTGTACGGCAGTG-3’  Reverse: 5’-GGCTGATCCCGTTGATTTCC-3’ |
| MUS TGFβ2 | Forward: 5’-CTTCGACGTGACAGACGCT-3’  Reverse: 5’-GCAGGGGCAGTGTAAACTTATT-3’ |
| MUS TGFβ3 | Forward: 5’-CAGGCCAGGGTAGTCAGAG-3’  Reverse: 5’-ATTTCCAGCCTAGATCCTGCC-3’ |
| MUS IL1β | Forward: 5’-CTGGAGAGTGTGGATCCCAAGCAA-3’  Reverse: 5’-GGGAACTCTGCAGACTCAAACTCCAC-3’ |
| MUS IL6 | Forward: 5’-AGGATACCACTCCCAACAGACCT-3’  Reverse: 5’-CAAGTGCATCATCGTTGTTCATAC-3’ |
| MUS IL10 | Forward: 5’-CTTACTGACTGGCATGAGGATCA-3’  Reverse: 5’-GCAGCTCTAGGAGCATGTGG-3’ |
| MUS TNFα | Forward: 5’-AAATGGGCTTTCCGAATTCA-3’  Reverse: 5’-CAGGGAAGAATCTGGAAAGGT-3’ |
| MUS cd11c | Forward: 5’-CTGGATAGCCTTTCTTCTGCTG-3’  Reverse: 5’-GCACACTGTGTCCGAACTCA-3’ |
| MUS iNOS | Forward: 5’-AGGGACAAGCCTACCCCTC-3’  Reverse: 5’-CTCATCTCCCGTCAGTTGGT-3’ |
| MUS STAB1 | Forward: 5’-ACGGGAAACTGCTTGATGTC-3’  Reverse: 5’-ACTCAGCGTCATGTTGTCCA-3’ |
| MUS Ym1 | Forward: 5’-GAAGGAGCCACTGAGGTCTG-3’  Reverse: 5’-GAGCCACTGAGCCTTCAAC-3’ |
| MUS Lyve1 | Forward: 5’-CTGGCTGTTTGCTACGTGAA-3’  Reverse: 5’-CATGAAACTTGCCTCGTGTG-3’ |
| MUS Fizz1 | Forward: 5’-GGAACTTCTTGCCAATCCAGC-3’  Reverse: 5’-AAGCCACAAGCACACCCAGT-3’ |
| MUS CD206 | Forward: 5’-CTCTGTTCAGCTATTGGACGC-3’  Reverse: 5’-CGGAATTTCTGGGATTCAGCTTC-3’ |
| MUS CD163 | Forward: 5’-ATGGGTGGACACAGAATGGTT-3’  Reverse: 5’-CAGGAGCGTTAGTGACAGCAG-3’ |
| **siRNA** | **Sequences** |
| Human siEZH2 | 5’-GCUGGAAUCAAAGGAUACA-3’ |
| Mus siEZH2-419 | 5’-GCACAAGUCAUCCCGUUAATT-3’ |
| Mus siEZH2-742 | 5’-GAAAGAUCUAGAGGAUAAUTT-3’ |
| Mus siEZH2-1377 | 5’-GCAAGUUCGAGAGCAUAAUTT-3’ |
| Negative control | 5′-UUCUCCGAACGUGUCACGU-3′ |
