# Supplementary material for: EZH2 suppression in glioblastoma shifts microglia toward M1 phenotype in tumor microenvironment
Source: J Neuroinflammation. 2017 Nov 13;14:220. doi: 10.1186/s12974-017-0993-4 (PMC5684749; doi:10.1186/s12974-017-0993-4)
Supplement: Supplementary file 2 — The KEGG and biological process enrichment analysis of EZH2-associated genes by microarray. Sample preparation: U87 cells were treated with siEZH2 and NC for 48 h at the concentration of 100 nM and then total RNA was extracted using a TRIzol reagent (Life Technologies, USA) according to the manufacturer’s instructions. The same samples at different times were repeated thrice and then these three copy samples were mixed for microarray using the Agilent Whole Human Genome Oligo Microarray kit, 4 × 44 K (Agilent Technologies, USA), consisting of approximately 41,000 genes and transcripts. The upper shows KEGG pathway and lower shows biological processes. (DOCX 130 kb) [file 12974_2017_993_MOESM2_ESM.docx]

**Additional file 2**: The KEGG and biological process enrichment analysis of EZH2-associated genes by microarray.


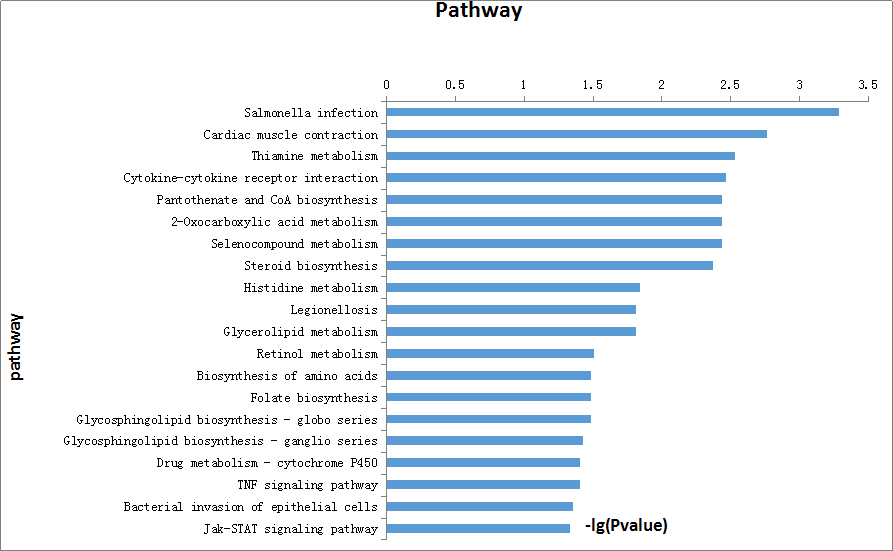


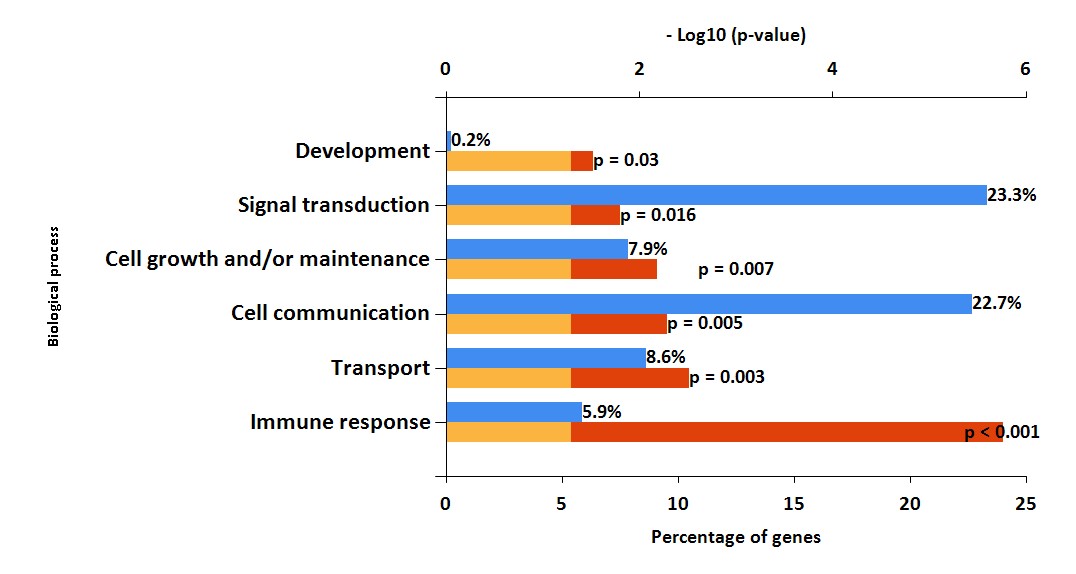


Sample preparation: U87 cells were treated with siEZH2 and NC for 48 h at the concentration of 100nM and then total RNA was extracted using a TRIzol reagent (Life Technologies, USA) according to the manufacturer's instructions. The same samples at different times were repeated thrice and then these three copy samples were mixed for microarray using the Agilent Whole Human Genome Oligo Microarray kit, 4 × 44K (Agilent Technologies, USA), consisting of approximately 41,000 genes and transcripts. The upper shows KEGG pathway and lower shows biological processes.
