# Supplementary material for: EZH2 suppression in glioblastoma shifts microglia toward M1 phenotype in tumor microenvironment
Source: J Neuroinflammation. 2017 Nov 13;14:220. doi: 10.1186/s12974-017-0993-4 (PMC5684749; doi:10.1186/s12974-017-0993-4)
Supplement: Supplementary file 3 — Expression levels of EZH2 mRNAs and proteins in GL261 glioma cells. The expression levels of EZH2 mRNAs and proteins were detected by real-time PCR and western blot in GL261 glioma cells treated with three kinds of siEZH2 and EZH2 functional inhibitor DZNep for 48 h. The working concentrations of siEZH2 and DZNep were 100 nM and 5 uM, respectively. Due to the potent inhibition on EZH2 expression, siR-419 was chosen for subsequent studies in GL261 glioma cell lines. (DOCX 162 kb) [file 12974_2017_993_MOESM3_ESM.docx]

**Additional file 3**: Expression levels of EZH2 mRNAs and proteins in GL261 glioma cells.


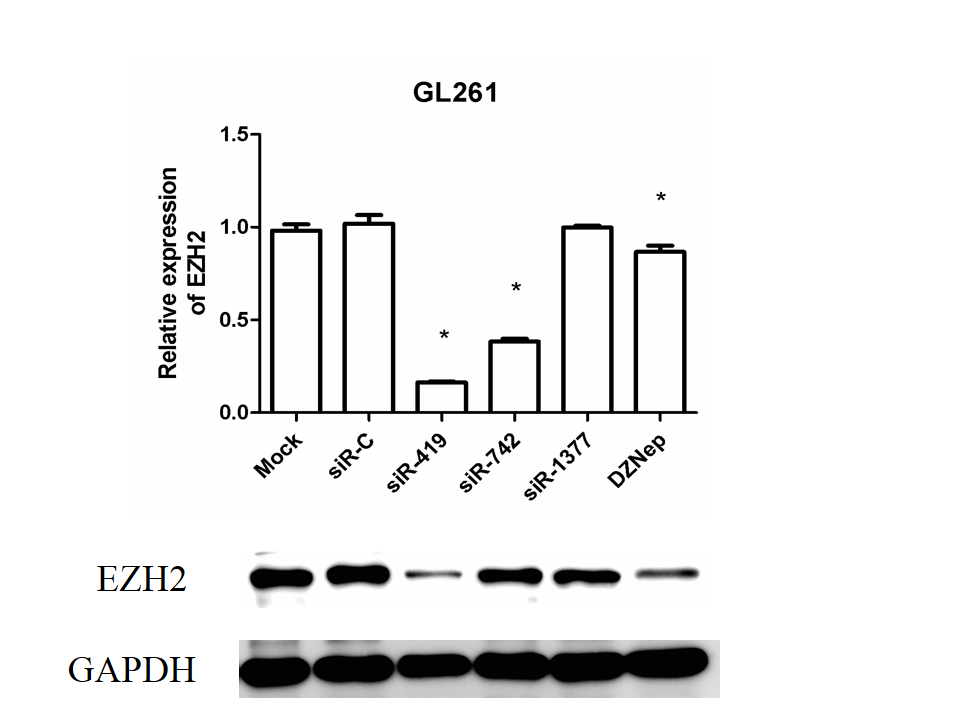


The expression levels of EZH2 mRNAs and proteins were detected by real-time PCR and western blot in GL261 glioma cells treated with three kinds of siEZH2 and EZH2 functional inhibitor DZNep for 48 hours. The working concentrations of siEZH2 and DZNep were 100nM and 5uM, respectively.

Due to the potent inhibition on EZH2 expression, siR-419 was chosen for subsequent studies in GL261 glioma cell lines.
